# Defective Interfering RNAs: Foes of Viruses and Friends of Virologists

**DOI:** 10.3390/v1030895

**Published:** 2009-11-10

**Authors:** Kunj B. Pathak, Peter D. Nagy

**Affiliations:** Department of Plant Pathology, University of Kentucky, Lexington, KY 40546, USA

**Keywords:** RNA virus, RNA structure, host factors, replication, recombination

## Abstract

Defective interfering (DI) RNAs are subviral RNAs produced during multiplication of RNA viruses by the error-prone viral replicase. DI-RNAs are parasitic RNAs that are derived from and associated with the parent virus, taking advantage of viral-coded protein factors for their multiplication. Recent advances in the field of DI RNA biology has led to a greater understanding about generation and evolution of DI-RNAs as well as the mechanism of symptom attenuation. Moreover, DI-RNAs are versatile tools in the hands of virologists and are used as less complex surrogate templates to understand the biology of their helper viruses. The ease of their genetic manipulation has resulted in rapid discoveries on *cis*-acting RNA replication elements required for replication and recombination. DI-RNAs have been further exploited to discover host factors that modulate *Tomato bushy stunt virus* replication, as well as viral RNA recombination. This review discusses the current models on generation and evolution of DI-RNAs, the roles of viral and host factors in DI-RNA replication, and the mechanisms of disease attenuation.

## 1. Introduction

Viruses are thought to be the ultimate parasites, multiplying inside the host cells and utilizing the resources of their hosts to produce vast number of infectious progenies. Intriguingly, many viruses have their own parasites, including defective interfering (DI) RNAs, as well as satellite viruses and satellite RNAs. For viruses with RNA genomes, the DI molecules consist of RNA sequences derived from the parent RNA virus, whereas the origin of satellite RNAs are not known in most cases. Both classes of subviral RNA are parasitic, since they have to use proteins coded by viruses, called helper viruses, for their replication inside the host cells. This review will focus on the DI-RNAs and their intimate relationship with their parent viruses and host cells.

DI-RNAs are created spontaneously during the replication of the viral genome and they multiply rapidly and eventually lead to slowing down of the parent virus’ multiplication [reviewed by 1,2]. These RNAs are called “defective” because they have lost the capacity to code for all the necessary viral proteins for independent replication and thus are defective in the absence of the parent (also called helper) virus. Accordingly, the helper virus is required to provide the missing replication protein(s) in *trans*. DI-RNAs are referred as “interfering” because they can attenuate the symptoms caused by the helper virus [reviewed by 2,3]. However, some defective RNAs do not interfere with multiplication of their helper viruses; in those cases they are simply called D-RNAs. In some cases, DI-RNAs can enhance the symptoms caused by their parent viruses. Importantly, DI-RNAs are distinct from other parasitic RNAs, called satellite (sat)RNA and satellite viruses, which are associated with helper viruses. The primary difference being that satRNAs do not show intensive sequence similarities with their helper viruses and the sources of their nucleic acid sequences remain uncertain [reviewed by 2]. The hierarchical game of parasitism does not stop here. To make the matter more complex, DI-RNA has been discovered even for a satellite virus [4]. This satellite derived DI-RNA strongly interfered with the parental *satellite pancium mosaic virus* (SPMV), which is one of the two sub-viral particles associated with *Panicum mosaic virus* (PMV). Thus, there is a unique complexity and dynamism in viral co-infections that include satellite RNAs, satellite viruses and DI-RNAs.

DI-RNAs are often observed during RNA virus infections of mammalian cell cultures when high multiplicity of infection is used [5]. DI-RNAs associated with plant virus infection has been mostly described from greenhouse samples or laboratory experiments [6–15]. DI-RNAs associated with viruses in the tombusvirus genus are among the most extensively studied. Also, the first DI-RNAs associated with a plant virus identified were derived from *Tomato bushy stunt virus* (TBSV) [11]. Thus, the TBSV DI-RNA will be discussed in detail as a model DI-RNA in this review.

There are several reviews [1–3,16,17], which comprehensively discuss the occurrence and genome structures of DI-RNAs. In this review, we will mainly focus on the current developments on different aspects of DI-RNAs and especially how they have shaped our understanding about the biology of parent virus infections.

## 2. Origin and Synthesis of Defective Interfering (DI) RNAs

DI-RNAs are synthesized by the viral RNA-dependent RNA polymerases (RdRp) that also replicate the parental virus genomes. Most of the DI-RNAs consist of non-contiguous portions of their helper virus’ genomes [16]. The popular model for DI-RNA formation is the viral polymerase driven template-switching mechanism [18].

### 2.1. Mechanisms of generation of DI-RNAs

#### 2.1.1. Replicase driven template-switching mechanism

RNA recombination plays a major role in producing DI-RNAs or defective RNA particles (*i.e.* packaged DI RNAs). The errors made by the RdRp, including template switching (also called replicase jumping), during the standard replication process of the viral genome is likely the main mechanism of genomic RNA-RNA recombination and also DI-RNA formation [18].

The replicase-driven template-switching model is supported by biochemical assays performed in cell-free systems with purified recombinant viral RdRps. The list includes two tombusviruses (TBSV and *Cucumber necrosis virus,* CNV), *Brome mosaic virus* (BMV), *Turnip crinkle virus* (TCV), *Cucumber mosaic virus*, *Bovine viral diarrhea virus*, and *Hepatitis C virus* [19–24]. The *in vitro* data from the above works suggested that breaks, strong hairpin structures, or AU-rich streches in the template (donor) RNA promotes the viral replicase to switch template to the acceptor RNA and then use the nascent RNA as a “primer” for resumption of RNA synthesis on the acceptor RNA. Interestingly, *cis*-acting sequences as well as sequence complementarity between the nascent RNA and the acceptor RNA may guide the template-switching events [18]. Primer extension experiments with RNAs representing the nascent strand revealed that rather short (2-to-5 nt) sequence complementarity between the primer and the acceptor template was sometimes sufficient to promote re-initiation of the replicase [20,21,25–27]. Additional works revealed recombination “cold-” and “hot-spots”, *i.e.* regions of decreased and increased recombination, respectively, in the BMV and TBSV genomes [20,21,28].

**Figure 1.**
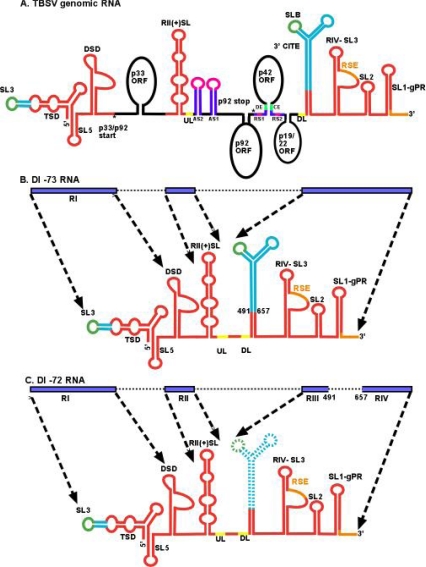
Genome and structural organization of TBSV genomic RNA and the prototypical DI-73 and DI-72 RNAs. (A) A cartoon showing structural details of the ∼4,800 nt TBSV genome (not to scale). The p33, p92^pol^, p41 and the overlapping p19/p22 ORFs are depicted as black ovals and labeled accordingly. Note that p92^pol^ overlaps with p33, sharing the same initiation codon. Sequences playing role(s) in translation, genome replication and sgRNA transcription are shown in turquoise blue, red and purple, respectively. Sequences involved in RNA-RNA interactions are shown in matching colors. Note that translation requires SL3-SLB interaction, UL-DL and RSE-gPR interactions are required for replicase assembly and AS1-RS1, AS2-RS2 and DE-CE interactions are crucial for sgRNA synthesis. (Abbreviations used are DSD: downstream domain; TSD: T-shape domain: RSE: replication silencer element; AS: activator sequence; RS: receptor sequence; CE: core element; DE: distal element; UL: upstream linker; DL: downstream linker; CITE: cap independent translation enhancer; SL: stem loop) [104] (B) Structure of the ∼800 nt DI-73 carrying three noncontiguous segments of the genomic RNA. Generation of DI-73 preserves critical replication elements (red) and the 3′CITE. The blue bars and dotted arrows depict the segments corresponding to genomic RNA. (C) Note that the other prototypical DI of ∼620 nt, named DI-72 RNA, has an additional deletion of the 3′CITE.

The formation of the prototypical TBSV DI-RNAs requires two or three recombination events, which likely occur sequentially [6]. For example, the TBSV-associated DI-72 RNA and other tombusvirus DI-RNAs are comprised of four noncontiguous RNA segments, namely Region I (derived from the 5′ untranslated region [UTR]), Region II (representing portion of the p92^pol^ ORF), Regions III and IV (mostly derived from the 3′ UTR) (Figure 1) [16,29]. Interestingly, the junction sites among the four noncontiguous regions in the above DI-RNAs do not show long sequence similarity. Therefore, it has been suggested that the recombination events during TBSV DI-RNA formation are unlikely to be random, but guided by *cis*-acting replication sequences [17].

#### 2.1.2. Forced template-switching mechanism

This model is a modified version of the replicase-driven template-switching model. The model postulates that the viral replicase switches template when it encounters the 5′ end of the template, which serves as a strong stop signal for the replicase. The 5′ end could be the natural end of the DI-RNA, so that recombination leads to the generation of head-to-tail DI-RNA dimers. New 5′ ends could also be generated via cleavages by host endo- and exoribonucleases, leading to partial degradation of the DI-RNA as demonstrated for the TBSV DI-RNAs [30–33]. These new 5′ ends of the partially degraded DI-RNAs can then become recombination hotspots [32,34]. These different types of recombination events could generate many different DI-RNA recombinants or promote the *de novo* formation of novel DI-RNAs. Due to the presence of large number of ribonucleases in host cells, this mechanism might be rather common for RNA viruses and DI-RNAs [35].

#### 2.1.3. RNA breakage and ligation mechanism

Another mechanism for RNA recombination based on RNA ligation has also been reported for Q-beta bacteriophage and poliovirus [36,37]. The major evidence provided for RNA breakage and transesterification was the interference with recombination when the 3′ OH group in the acceptor strand was altered to inhibit ligation. It is yet to be seen if this mechanism is involved in DI-RNA formation.

### 2.2. Viral replication proteins as factors influencing the formation/accumulation of DI-RNAs

**Figure 2.**
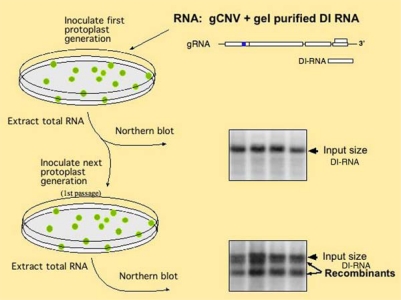
Experimental scheme to test DI-RNA evolution in plant protoplasts. Generation of DI-RNA recombinants are tested via using the total RNA extract from the first protoplast samples for electroporation of the second batch of protoplasts (1st passage). The images on the right show Northern blot analysis of the total RNA extracts used to detect the original DI-RNA and the recombinants [41,42].

Evidence to support the roles of replication proteins in DI-RNA formation has been obtained with the helicase-like protein 1a of BMV. Mutations within the 1a protein altered the sites of RNA recombination when compared with BMV infections containing the wt 1a [38]. In addition, mutational studies on BMV 2a polymerase also indicated the replicase’s role in recombination [39]. A 2a mutation affected the precision as well as the location of RNA recombination sites. Similarly, mutation in the polymerase gene of influenza virus, led to an increase in the synthesis of DI-RNAs [40]. The authors proposed that the mutation destabilized the polymerase-viral RNA complex during the elongation step. A role for the replicase in RNA recombination was further bolstered by studies on the p33 auxiliary replication protein for CNV [41]. Frequency of recombination for tombusvirus DI-RNA was affected by mutations in the RNA binding (RPR) domain of p33 (Figure 2). Two p33 mutants tested enhanced the recombination and 5 of the 17 mutants tested slowed down the accumulation of recombinants. Interestingly, the recombination promoting mutants also increased the level of subgenomic RNA transcription [42]. The connection between transcription and recombination is not that surprising as both processes utilize the viral replicase complex. Mutations within the N-terminal portion of p92^pol^ polymerase, which overlaps with p33, did not have similar affects on recombination, suggesting different roles for RPR in p33 and p92^pol^ proteins with respect to RNA recombination. How these mutations affect recombination, or how they enhance template-switching remains to be tested.

Most plant RNA viruses code for two or more replication proteins and the amount and the ratio of the replication proteins could be another factor affecting RNA recombination and DI-RNA formation. This was tested with the tombusvirus p92^pol^ and p33 replication proteins in yeast, a surrogate model host [43]. High level of p33 was shown to increase the accumulation of recombinant DI-RNA. The effect on recombination was even more profound when p92^pol^ was expressed at a high level in yeast. In addition, the ratio of p33 and p92^pol^ also affected recombinant DI-RNA formation. It is possible that too high a level of p92^pol^ RdRp makes the viral replicase complexes less precise and more prone to template switching.

### 2.3. Viral RNA elements as recombination “hot spots” during DI-RNA formation

The role of *cis*-acting replication elements in RNA recombination has been proposed before in several viral systems [18], including the replication enhancer element of TCV and TBSV, the subgenomic promoter region of BMV and RII sequence of TBSV that binds to the replication proteins [20,23,24,44]. The *cis*-acting replication elements likely promote the formation of DI-RNAs as well, as shown for the RIII enhancer element and for RII that is required for RNA recruitment during TBSV replication [20,25,45,46]. The DI-RNAs containing duplicates or triplicates of RIII, recombined at much higher rates in *N. benthamiana* protoplasts [41]. The recombination junctions were imprecise and clustered around RIII, suggesting RIII acted as a recombination “hot spot”.

Additional highly active “hot-spot” regions for RNA recombination that might also promote DI-RNA formation consist of AU-rich stretches or stable secondary structures [47,48]. Insertion of a short AU-rich stretch into DI-72, a model template for TBSV, rendered this RNA highly recombinogenic [49]. Comparable AU-rich stretches in BMV and Human immunodeficiency virus, a retrovirus, also promoted recombination [47,50,51]. In contrast, GC-rich sequences in BMV acted as recombination “cold spots” [52]. The similar insertion of a GC-rich sequence in TBSV DI-RNA did not delay or inhibit the accumulation of recombinants [49], suggesting that some RNA sequences are utilized with different efficiencies by the BMV and TBSV replicases.

Strong RNA secondary structures in templates can also serve as recombination hot spots. These structures might form between two viral RNAs containing short complementary sequences, leading to the formation of a heteroduplex [48]. Strong hairpins also exist in viral RNAs and they might promote RNA recombination. Experimental evidence supporting the role of hairpin structures in DI-RNA evolution was obtained with *Cymbidium ringspot virus* (CymRSV) [53].

Hairpin structures serve frequently as *cis*-acting replication elements that bind to viral or host proteins. For example, region II (RII) in TBSV is a recombination hot spot [20,32], which is likely due to its ability to bind to the p33 replication protein [54]. Another factor that could make RII a recombination hot spot is the presence of the strong RII(+)-SL hairpin that acts as a road block for the host Xrn1p, a 5′-3′ exoribonuclease. This in turn, creates partially degraded viral RNAs that can serve as efficient substrates for RNA recombination, and also promote DI-RNA formation [24,30,32,33].

### 2.4. The effect of host factors on DI-RNA formation

In spite of significance in virus replication and possibly DI-RNA formation, our understanding of the role of the hosts in RNA recombination is rather limited. Significant progress has been made by establishing yeast as a model host for TBSV replication/recombination [55]. Serviene and co-workers screened the yeast single gene knock out library for the effect of individual gene deletions on the accumulation of recombinant DI-RNAs [33] and identified 11 host genes that promoted/modified viral recombinant accumulation, while 5 host genes were found to act as TBSV recombination suppressors. Additional work on one of the identified host factors, namely *XRN1* 5′ to 3′ exoribonuclease involved in the RNA degradation pathway, led to the discovery of partial TBSV RNA degradation products in *xrn1Δ* yeast and *xrn4*-silenced *N. benthamiana* plants [30–33]. The partial TBSV RNA degradation products served as recombination substrates, promoting the formation of novel DI-RNA molecules. *In vitro* data using purified Xrn1p, as well as *in vivo* work of overexpression of this exoribonuclease demonstrated that Xrn1p works as a suppressor of TBSV RNA recombination [32]. Moreover, expression of the *Arabidopsis thaliana* AtXrn4p, which is an orthologue of the yeast Xrn1p in *Nicotiana benthamiana,* led to degradation of viral RNAs (Cheng *et al.* 2007). In agreement with the yeast data, silencing of *XRN4* in *N. benthamiana* led to higher accumulation of the DI-RNA recombinants (Jaag and Nagy 2009).

The screening of host genes for TBSV DI-RNA recombination was further extended to 800 essential yeast genes [56]. The authors reported the discovery of 16 genes modulating the accumulation of DI-RNA recombinants. Studies showed that down regulation of these host genes affected the recombination in different ways. For example, (i) some host genes changed the ratio of replication proteins p92^pol^ and p33 in yeast, which affected DI-RNA formation [43]; (ii) others altered the stability of DI-72; and (iii) some affected the replication of the template. It is important to note that in the above reports, *de novo* generation of DI-RNAs from the parent virus was not studied, instead, already generated DI-RNA was used as a model template.

Another example of the host’s role in generation of DI-RNA is the demonstrated requirement of host dicer in production of DI-RNAs [35]. The authors found that dcl-2 (the host dicer gene) mediated the generation of DI-RNA associated with a hypovirus infecting *Cryphonectria parasitica,* the chestnut blight fungus. Moreover, in *Δdcl-2* strain, the absence of DI-RNA indicated that replicase errors might not be the only factors affecting the generation/accumulation of DI-RNAs [35].

### 2.5. Environmental and other factors affecting the formation of DI-RNAs

Apart from the roles of viral replicase proteins and the replication/recombination elements present in the viral genomic RNA, host specificity and growth conditions have also been implicated to affect the synthesis DI-RNAs [57]. For example, DI-RNAs associated with *Broad bean mottle virus* (BBMV) infection were observed in some host plants even when infections were started with low multiplicity of inocula (MOI), whereas in other plants, high MOI for several passages were needed to generate DI-RNAs. Interestingly, the sizes of the DI-RNAs as well as the rate of their accumulation varied with the growth temperature. It is not yet known whether these changes are because of some change in selection pressures on DI-RNA accumulation (i) due to variable activities of the host defense mechanisms, such as gene silencing; (ii) due to altered RdRp activities, such as template-switching, under various conditions; or (iii) due to changes in the structure of the viral RNA. We also cannot rule out the combination of all the above factors.

The important role of the host for TBSV DI-RNA accumulation was further supported by an altered rate of DI-RNA accumulation in *N. benthamiana* and pepper (*Capsicum annuum*) [58]. In *N. benthamiana*, DI-RNA accumulation was easily detectable and able to attenuate the symptoms, whereas, in pepper, even continuous virus passages failed to generate detectable levels of DI-RNA. Moreover, the pepper plants showed severe local and systemic chlorosis. Thus, the results from *N. benthamiana* indicates that DI-RNA formation is a way for plants to attenuate virus-induced symptoms.

## 3. Continuous Evolution of DI-RNAs

Most RNA viruses undergo rapid evolution due to their error prone RdRps [59–61]. Mutations and RNA recombination are the two main mechanisms that drive viral RNA evolution. Between these two mechanisms, the latter produces more dramatic changes by covalently joining two pieces of noncontiguous RNA. As DI-RNAs share the replicase with their helper viruses and have RNA elements derived from their parent viruses, they likely undergo similar evolutionary process and mechanisms. The rate of evolution of DI-RNAs, however, is likely to be faster than their helper viruses due to the higher genetic plasticity of DI-RNA genomes, resulting from reduced purifying selection pressure, which effectively eliminates nonfunctional viral genomes.

### 3.1. Deletion

DI-RNAs are mainly formed via RNA recombination/genome rearrangements. It is likely that, even though they are more robust, only a fraction of DI-RNAs formed will be replication competent and able to compete in plants. The replication competent DI-RNAs have to compete with the helper virus for the viral RdRp and with other DI-RNAs, resulting in continuous DI-RNA evolution under high selection pressure. DI-RNAs with mosaic genomes, such as the TBSV-associated DI-RNAs (Figure 1) are formed in a step-wise deletion fashion, followed by additional changes to tailor their sizes and RNA structures until they become maximally competitive under that particular environment/selection pressure [6]. The authors suggested that the primary driving force in DI-RNA evolution is the replication efficiency of the DI genome. One of the classical examples is the evolution of DI-73 RNA into DI-72 RNA by the deletion of sequences between RIII and RIV (Figure 1). The deleted region, designated as R3.5, contains a Y-shaped structure that acts as a translation enhancer in the TBSV genomic RNA (Figure 1). It is likely that this region interacts with translational factors and promotes translation of viral protein. This, in turn, would lead to competition between translation and replication, which are mutually exclusive processes. Accordingly, DI-73 RNA would need to be rescued from translation or stripped from bound translation factors prior to entering replication. This step would make DI-73 less efficient in DI-RNA recruitment into replication. In contrast, DI-72 and other DI-RNAs lacking R3.5 sequences do not face this competing process and can directly enter replication. This difference likely explains why DI-73 evolves to DI-72 over time [6].

### 3.2. Duplication and nucleotide insertions

Albeit sequence deletions and rearrangements are the most common events during DI-RNA formation, additional genetic changes also occur frequently. For example, DI-RNAs can undergo other types recombination events, such as segment duplication or nucleotide insertions that could enhance their competitiveness [62,63]. By using DI-73, a prototypical TBSV DI- RNA, the authors in this study showed that after serial passaging for 10 times in protoplasts, DI-73 evolved to a shorter DI-RNA with structures similar to DI-72 RNA. However, in one of the cases, DI-73 evolved to a longer DI-RNA, designated as DI-R1, containing duplication of a 130 nt sequence derived from RII in the 6th passage. But by 9th passage DI-R1 was no longer detectable and was replaced by a shorter DI-RNA, similar to DI-72 in size (termed DI-B103). Competition assays using co-inoculation of these DI-RNAs in the presence of the helper virus in cucumber protoplasts revealed that DI-R1 was more competitive than DI-73 or DI-72 RNAs. It is likely that duplication of the critical RII sequence involved in RNA recruitment for replication [54] provided an extra *cis*-element for binding to the viral p33/p92^pol^ replicase proteins. This in turn, could result in enhanced viral RNA recruitment in case of DI-R1. A single U insertion in RII was found to make DI-B103, containing only one RII, more competitive than DI-R1 containing two wt RIIs. This insertion may have made the single RII in DI-B103 more efficient at binding p33, however this idea remains to be tested.

Competition for limited viral- and host resources drives the evolution of DI-RNAs at faster rates than that of their parental virus. Accordingly, we observed rapid evolution with a minimal DI-RNA carrying only a minimal set of essential *cis*-acting elements required for the assembly of replicase complex. This minimal DI-RNA goes through rapid evolution in yeast and in plants by sequence duplication and even triplication. (Pathak KB, Panaviene, Z. and Nagy, PD, unpublished data). The presence of multiple *cis*-acting elements may help the new DI-RNAs to increase binding to more replicase proteins cooperatively or facilitating fast recruitment for replication. It is interesting to note that too strong competition with the helper virus for the replicase complex could be counterproductive for the DI-RNA, since then this leads to reduced number of helper virus genomes per cell, followed by reduction in the synthesis of viral replication proteins. This in turn, limits further replication of the DI-RNAs. Therefore, it is likely that the best adapted DI-RNAs are capable of compromise, allowing the helper virus to replicate as well. Many RNA viruses also utilize “*cis*-preferential” replication [42,64–66] where the viral RNA that produces one of the replication proteins has an advantage for replication, likely due to more efficient binding to the replication protein. In contrast, *trans*-replicating DI-RNAs have to “steal” the replication proteins from a pool of newly synthesized proteins.

Nevertheless, their small size and limited or no contribution to expression of functional proteins give DI-RNAs a major advantage over their helper virus. In many cases, this results in attenuation of disease symptoms caused by the helper virus. In order to systemically infect the host plants, the DI-RNAs also have to move from cell to cell via plasmodesmata. If viral movement proteins bind cooperatively to the viral RNA, DI-RNAs would be at a disadvantage, as they have short genomes. To circumvent this problem, DI-RNAs might generate head-to-tail dimers [67]. In this study the authors reported more cell-to-cell movement for head-to-tail dimers of CymRSV DI-RNAs when compared with monomers. However, in protoplasts, where cell-to-cell movement does not occur, accumulation of DI-RNA monomers and dimers took place at similar levels.

## 4. The Mechanism of Interference by DI-RNA

Those DI-RNAs that maintain critical *cis*-acting replication elements are usually excellent templates during replication due to: (i) their small sizes; (ii) being efficient RdRp substrates; (iii) and lacking competition between translation and replication processes for the RNA templates in case of translation-incompetent DI-RNAs. Therefore, in many cases the accumulation of the helper virus is inhibited by the most competitive DI-RNAs, resulting in the symptom attenuation in host plants. However, the interaction between the DI-RNA, helper virus and the host is more complex, due to the effect of DI-RNAs on induction of host defense responses or sequestering resources of the host cells by the DI-RNA. Accordingly, DI-RNAs of BBMV and TCV enhance the symptoms caused by their helper viruses [reviewed by 2]. Scientists are still in quest to identify the mechanisms of the above-discussed effects. The mechanisms of interference by DI-RNAs fall into three major categories [reviewed by 2].

### 4.1. Competition for viral- and host resources

In the absence of DI-RNAs, the pool of replication proteins, host factors, nucleotides, host membranes and all other factors is utilized entirely by the helper virus for its own multiplication. Once DI-RNAs are in the picture, the above factors then have to be shared by both the viral and sub-viral RNAs. Eventually, the helper virus is out-multiplied by the more competitive DI-RNAs. This type of helper virus inhibition by DI-RNA is known to be dose-dependent. The co-infection experiments done in *N. benthamiana* protoplasts with TBSV and DI-RNAs, showed 65% suppression of TBSV genomic RNA accumulation when equimolar amount of helper versus DI-RNAs were used [68].

Additional findings, like inability to attenuate the symptoms by some DI-RNAs that accumulate to high levels [29], and host specific accumulation of DI-RNAs [69] further indicate that the effect of DI-RNAs on the helper virus accumulation involves complex mechanisms in addition to simple competition with the helper virus.

### 4.2. Modulation of the functions of viral factors

Several studies on the attenuation effects of DI-RNAs on the symptoms caused by the helper virus did not fit into the simple model of competition desribed above. For example, when *N. benthamiana* plants were co-infected with TBSV genomic transcripts and DI-RNAs, the reduction in viral genomic RNA replication proteins accumulation was less dramatic than the reduction in the levels of p19 suppressor of gene silencing and p22 movement proteins along with the levels of subgenomic (sg) RNA2 [70]. Not only the amounts of these viral factors were decreased but also the spatial and temporal distribution was affected, suggesting that the DI-RNA preferentially interfered with the production and/or translation of sgRNA2.

### 4.3. DI-RNA-triggered gene silencing response of the host

RNA interference (RNAi) or posttranscriptional gene silencing (PTGS) is a eukaryotic cellular response to the presence of double-stranded (ds) RNAs. These dsRNAs can be either endogenous or parasitic in nature [reviewed by 71,72]. The mechanism can be used to either regulate gene expression or to mediate resistance/host response against pathogens. Plants use this mechanism as a major antiviral strategy [reviewed by 72,73]. The host RNAi machinery use 21–25 nt virus derived small interfering (si) RNAs to guide PTGS against viral genomic RNAs. However, viruses fight back by avoiding or delaying recognition by host surveillance due to the formation of replicase complexes hidden in host membranes and by expressing suppressors of gene silencing. For example, p19 of tombusviruses has been shown to be a potent suppressor of gene silencing [74]. Therefore, DI-RNA associated interference with the parent virus replication could be due to hindrance with the activity of p19 [75]. The authors saw a decrease in accumulation of p19 upon co-infection with tombusviruses and DI-RNAs. Moreover, the presence of DI-RNAs enhanced the generation of virus-specific siRNAs, leading to accumulation of free siRNAs unbound by p19. These free siRNAs could thus trigger robust PTGS against the helper virus. Evidence supporting this mechanism is the observation that PTGS is impaired at low temperatures and the DI-RNA-mediated attenuation of the helper virus was also hindered at low temperature.

In another study, a more direct role for DI-RNA in triggering gene silencing against its own helper virus emerged. It was shown by Szittya and colleagues [76] that the replication of CymRSV, a tombusvirus, in *N. benthamiana* plants triggered PTGS, resulting in the accumulation of siRNAs corresponding to the CymRSV sequence. They further showed that the PTGS machinery did not efficiently degrade the shorter DI-RNAs. Moreover, this defect was not associated with the RNase complex (RISC) because the system could efficiently silence the larger DI-RNAs and a different helper virus. The length of target molecule was not a deciding factor in this target activity, rather specific sequences and structures of the DI-RNAs played a major role in PTGS. Thus, DI-RNAs could trigger potent gene silencing response against the helper virus without hurting themselves from the same response. In a further attempt to characterize DI-RNA associated symptom attenuation, sequence elements of tombusvirus-associated DI-RNAs were identified [77,78].

The more we will learn about viral pathogenesis and the interaction and competition between DI-RNA and the helper virus, more we can focus our research to dissect DI-RNA mediated attenuation in molecular mechanistic term. It is possible that DI-RNA can modulate interaction between two or more viral proteins or affect their posttranslational modifications. It is also not unrealistic to imagine that DI-RNAs can sequester important host factor(s) away from helper virus replication and thus, reducing helper virus accumulation and moderating the symptoms associated with helper virus infections.

## 5. DI-RNA as a Tool in Virology and Biotechnology Applications

### 5.1. DI-RNAs as vectors to express recombinant proteins in plants

The use of plant viruses for recombinant protein production is widespread in research and growing for industrial production of useful proteins. In recent years, we have seen advances in both development of first-generation (based on ‘full virus’) vectors and second-generation (‘deconstructed virus’) vectors [reviewed by 79]. To this end, uses of DI-RNA sequence based constructs are also getting popular both for gene silencing [80] and expression of heterologous proteins [81,82] . The use of DI-RNAs as vectors can be more flexible in their genetic manipulations because they are not required for infection process and are subjected to less stringent selection pressure [83].

### 5.2. DI-RNAs as surrogate templates to study virus replication

DI-RNAs are versatile tools at virologists’ disposal. DI-RNAs often multiply at higher rates, are smaller than the full genome of the virus and contain the *cis*-acting replication elements for replication and other steps in viral multiplication cycle. Since many DI-RNAs do not code for proteins, mutational studies on DI-RNAs make the results easier to interpret due to separation of *trans*-acting protein factors and *cis*-acting RNA elements. Thus, critical *cis*-acting replication elements important for different steps in viral replication can be deductively discovered. These RNAs also are adapted to use *trans* (viral and possibly other) factors for their replication enabling a more experimental control over those factors so that a virologist can regulate them spatially and/or temporally. Indeed, using DI-RNAs combined with other modern tools, many *cis*-acting RNA elements for tombusviruses were deciphered [reviewed by 17,84]. DI-RNAs also proved to be useful in discovering host factors modulating viral replication. To this end, a yeast system using TBSV DI-72 as an experimental replicon (rep)RNA was developed [55,85]. In the above yeast system, the repRNA works as an independent replicon, capable of assembling the viral replicase complex and performing most of the steps in replication in the absence of a helper virus. Based on the yeast/TBSV repRNA system, genome-wide and proteomics-based screens have been performed [33,56,86–90]. This in turn, accelerated the identification of more than hundred host factors affecting TBSV replication and ∼40 host factors affecting RNA recombination. In addition, the system is also useful in dissection of the mechanisms by which these host factors affect viral replication and RNA recombination. To better explain the roles of *cis*-acting replication elements, viral and host factors discovered with the assistance of DI-RNA, we divide the replication cycle of the plus stranded RNA viruses further into different steps in chronological order [reviewed by 91,92].

### 5.3. Template selection and DI-RNA recruitment into replication

Similar to the viral genomic RNA, DI-RNAs should also be recognized selectively from the large pool of host RNAs. The selection of the viral RNA requires specific interaction with viral- or host proteins. For TBSV DI-RNAs, the selective RNA recognition is mediated by the cytosol-exposed C-terminal portion of p33 replication protein in yeast [54,93]. Detailed mutational studies on DI-RNA revealed the essential role for a C•C mismatch within the RII(+)-SL sequence (Figure 1) in binding to p33 [54]. When the C•C mismatch was mutated to G=C, then binding of DI-72 to p33 was lost and this DI-RNA was unable to replicate in the yeast system or the full-length TBSV genomic RNA carrying the comparable mutation in *N. benthamiana* plants [54,93]. Thus, the C•C mismatch sequence can be regarded as the “identity card” of the virus, allowing the viral replicase complex to achieve high selectivity to replicate only viral RNA. As expected, the C•C mismatch region is also required for replication of DI-72 repRNA in a yeast extract capable of supporting one full cycle of replication *in vitro* [24,94].

**Figure 3.**
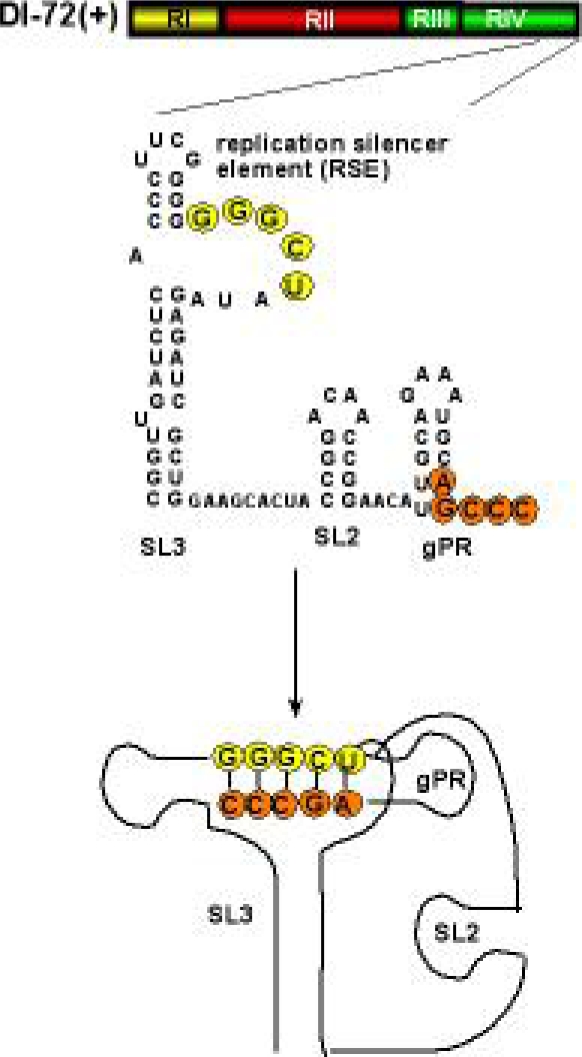
The predicted secondary and tertiary structure of the 3′ UTR in DI-72(+) RNA. A 5 nt base-pairing between RSE in the internal loop sequence of SL3 and gPR stabilizes the tertiary structure. This interaction is critical for the assembly of the functional TBSV replicase [95,112].

In addition to interacting with the viral replication proteins, DI-RNA is likely bound by select host proteins. To identify the host proteins that bind viral RNA, TBSV DI-72 (+)RNA was used as a probe to screen RNA binding proteins in a yeast proteome-wide chip carrying 4,100 purified yeast proteins [86]. Five of the identified host proteins in the above screen were further confirmed with other approaches, e.g. gel-shift and pull-down assays. One of the factors discovered was translation elongation factor eEF1A. This protein also co-purified with the tombusvirus replicase. Further studies showed that eEF1A binds to the silencer sequence present in the 3′UTR (Figure 3) [86], which is required for the assembly of the tombusvirus replicase complex [95,96]. A mutation affecting guanine exchange factor (GEF) requirement of eEF1A inhibited DI-72 repRNA accumulation in yeast. It has been proposed that eEF1A interaction with DI-72 RNA might be needed for RNA recruitment into replicase complex or viral RNA synthesis (Z. Li and P. D. Nagy, unpublished).

Another host factor that exerts its effect on viral replication directly via binding to the viral RNA is Nsr1p (also known as nucleolin). This factor was discovered during screening of the yeast knock out (YKO) library for TBSV replication [90]. Virus replication was boosted three folds in the absence of NSR1. Further analysis revealed that Nsr1p binds to RIII in DI-72 (+)RNA (Jiang, Li and Nagy 2009; in press). Indeed the Nsr1p mediated inhibitory effect on DI-72 repRNA accumulation *in vivo* was lost when DI-72 repRNA missing RIII, the target for Nsr1 binding, was used as a replicon RNA in above study. This protein may inhibit TBSV replication via specific binding to the viral RNA and, thus, resulting in inefficient repRNA recruitment for replication.

After the RNA has been selected for replication, the viral proteins and RNA complex has to be transported to the site of replication. TBSV assembles the replicase complex on the cytosolic surface of peroxisomal membranes [97–99]. It has recently been shown that the host shuttle protein Pex19p, which is involved in peroxisomal membrane protein transport, play a role in TBSV protein transportation to the site of replication [100]. Pex19p binds to the peroxisomal targeting signals of p33 *in vitro* and is also associated with the replicase complex, albeit only temporarily. When Pex19p was mistargeted to mitochondrial membranes, the wt p33 also ended on the same mitochondrial membranes. Interestingly, viral replication as well as the replicase activity was inhibited as a result of Pex19p mislocalization to the mitochondrial membranes.

Another host protein involved in localization/transportation of the viral replication proteins is the heat shock protein 70 (Hsp70). Using a temperature-sensitive mutant of Hsp70 at nonpermissive temperature has led to cytosolic localization of p33 replication protein [101]. Shifting down to permissive temperature resulted in re-localization of p33 to the peroxisome membrane surface in yeast.

Overall, the above studies revealed that both viral- and host proteins are involved in RNA template selection and the recruitment of the viral RNA/p33/p92^pol^ complex to the site of replication.

### 5.4. Factors affecting the assembly of the viral replicase complex

The assembly of the viral replicase complex is still a poorly understood process. Interestingly, the viral RNA plays an essential role in the replicase assembly both *in vivo* and *in vitro* [94,96,102,103]. Detailed work with TBSV RNA revealed that RII(+) (Figure 1) is also crucial for the assembly of tombusvirus replicase complex in yeast [96]. In this study, authors have defined the minimal *cis*-acting elements required for the assembly of the tombusvirus replicase complex. The affinity purified viral replicase complex was active on external templates only when the full-length DI-72 repRNA was co-expressed with the p33 and p92^pol^ replication proteins in yeast. The minimal repRNA still capable of supporting the assembly of the replicase complex consisted of RII(+)-SL hairpin, the replication silencer element and genomic promoter (Figure 1) [96]. The exact role of the viral RNA in the assembly process is currently under heavy investigation. The viral RNA might provide an assembly platform to bring together the viral and host proteins. The RNA may also play a role in making structural change(s) in the viral RdRp required for activation of the polymerase function of the RdRp. Recently long-range RNA interacting elements UL and DL in the TBSV genomic RNA have been discovered that play a role in replicase assembly [104]. Long distance base pairing between UL and DL sequences juxtapose RII(+)-SL and the replication silencer and gPR within the 3′-UTR (Figure 1). These latter two elements had already been shown to be important in replicase assembly [94,96]. It is interesting to note that UL-DL interaction is not crucial for replicase assembly in DI-73, probably because RII(+)-SL and the 3′-UTR are already in close proximity (Figure 1B), unlike in the genomic RNA, where they are 3000 nt apart (Figure 1A).

Both viral replication proteins of TBSV are essential for the assembly of the functional virus replicase [94,102]. Interestingly, the p33:p33/p92^pol^ interaction domains in these replication proteins seem to be critical for the assembly, suggesting that these proteins are participating in multimeric complex formation, which likely needed for the formation of membrane invaginations, called spherules. These spherules are the predicted structures supporting TBSV replication [98].

In addition to the viral RNA and viral replication proteins, host factors play a role in tombusvirus replicase assembly. The best-characterized host factor in the replicase assembly process is Hsp70. Proteomics analysis of the tombusvirus replicase complex revealed the presence of Hsp70 and 5–10 other host proteins within the replicase complex [86–88]. *In vitro* work indicated that Hsp70 plays a role in membrane insertion of the viral replication proteins [105]. The essential role of Hsp70 in the assembly of the TBSV replicase was confirmed by Pogany and colleagues, using a yeast extract depleted in Hsp70 [94]. The addition of purified recombinant Hsp70 to the above cell-free assay complemented the defect, leading to the assembly of the viral replicase complex and active replication of the DI-72 (+)repRNA *in vitro* [94].

### 5.5. Factors affecting the replication of DI-RNA

After the assembly of the replicase complex is finished, the synthesis of the complementary (−)-strand from the plus (+)-stranded DI-RNA takes place. The newly made (−)-strand then serve as a template for synthesis of new (+)DI-RNAs. The replication process of (+)-stranded RNA viruses and associated DI-RNAs is asymmetrical, leading to 20–100-fold more copies of (+)RNAs than (−)-strand RNA. One of the major factors in regulation of DI-RNA replication is the DI-RNA carrying *cis*-acting elements in both strands [reviewed by 17]. The *cis*-acting elements include the genomic promoter (gPR) in the 3′ end of (+)DI-RNA [106], a complementary promoter element (cPR) in the 3′ end of (−)RNA [46] and replication enhancer elements in RI(−) and RIII(−) [107]. An intriguing replication modulator element is the replication silencer (Figure 3), which is involved in the assembly of the replicase complex and possibly in the regulation of the (–)-strand synthesis [95]. This element interacts with gPR, making the promoter recognition by the RdRp weaker *in vitro*. It is currently under investigation how this interaction is regulated and what viral- or host proteins are involved in “unsilencing” this interaction.

The asymmetrical RNA synthesis is affected not only by *cis*-acting RNA replication elements, but host factors as well. One such host factor is a metabolic enzyme called glyceraldehyde-3-phosphate dehydrogenase (GAPDH), which was discovered as a component of the tombusvirus replicase complex via a proteomics analysis of a purified viral replicase preparation [88]. When TBSV DI-72 RNA was replicated in yeast cells, the cellular distribution for GAPDH changed dramatically due to re-localization from the cytosol to the site of replication (peroxisome) [108]. Down-regulation of GAPDH levels in yeast correlated with reduced level of (+)-strand DI repRNA. GAPDH was shown to bind to (–)-strand of DI-72 repRNA via an AU pentamer sequence. It was proposed that the role of GAPDH is to retain the (–)-strand repRNA intermediate in the replicase complex, thus facilitating asymmetrical replication. The data from the yeast host were also validated in *N. benthamiana* host [108].

### 5.6. Release of (+)DI-RNA progeny from the replicase complex and disassembly of replicase complex

**Figure 4.**
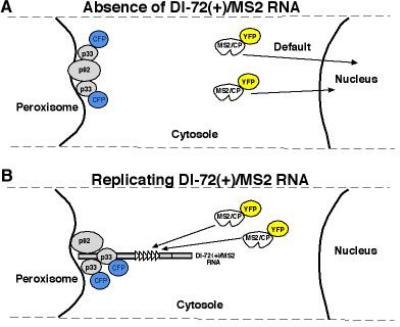

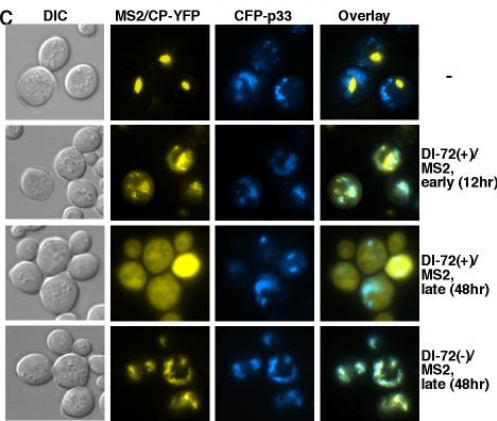
Different subcellular localization of plus- and minus-stranded DI-72 RNA during replication in yeast. (A) Schematic presentation of subcellular localization of MS2/CP-YFP and CFP-p33 in yeast in the absence of viral RNA and (B) in the presence of DI-72(+)/MS2. Specific interaction between the MS2 CP and the MS2 CP recognition hairpin (present in six copies in DI-72(+)/MS2) should result in re-localization of MS2 CP as shown. (C) Co-localization of CFP-tagged p33 and the YFP-tagged MS2/CP bound to the DI-72(+)/MS2 RNA in yeast cells using epifluorescence microscopy. Note that DI-72(–)/MS2 RNA (bottom panel) contains the six copies of MS2 CP recognition hairpins in complementary orientation. Therefore, MS2/CP-YFP could only bind to the negative-stranded DI RNA, which is generated during the replication of DI-72(–)/MS2 RNA.

After the synthesis of the new (+)DI-RNA progeny by the replicase, (+)RNA is released to the cytosol (Figure 4B), while the (–)RNA intermediate is kept within the complex most or all the time (Figure 4C) [97].

**Figure 5.**
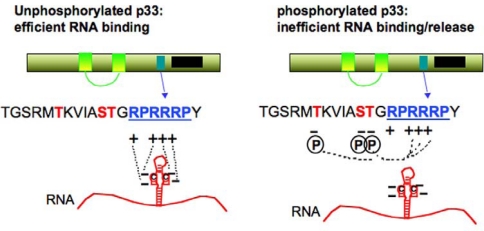
Schematic presentation of the phosphorylation sites in the TBSV p33 replication protein. The phosphorylated aminoacids are shown in red, while the RNA binding site is indicated with blue letters. The negative charges of phosphorylated amino acids and the positive charges of arginines are shown. Possible interactions are depicted with dotted lines. Note that the RNA has also negative charge, resulting its release from p33 after phosphorylation of p33 as shown [109].

So far host factors and viral RNA sequences have not been discovered playing role(s) in the release of (+)RNA progeny. However, viral protein modification has been proposed to modulate this process [109]. This is based on the observation that p33 gets phosphorylated *in vivo* and *in vitro* as well [109,110]. The phosphorylated form of p33 lost its ability to bind to (+)DI-72 RNA associated with TBSV and *in vitro* phosphorylation of the p33:DI-72 RNA complex led to the release of the RNA from the complex [109]. In contrast, p33 mutants mimicking the unphosphorylated stage of the protein bound efficiently to DI-72 RNA, suggesting that phosphorylation/unphosphorylation of p33 might regulate the release of (+)RNA from the replicase complex (Figure 5).

Roles of other host proteins in viral protein modification have also been demonstrated. A host ubiquitin conjugating enzyme Cdc34p as well as Rsp5p ubiquitin ligase have been shown to ubiquitinate p33 *in vitro* [87,111]. However, the actual role of p33 ubiquitination is currently unknown. It is possible that this protein modification modulates the viral protein-protein or viral protein-host protein interactions important for viral replication.

Altogether, the use of DI-RNA as surrogate template contributed a great deal to our understanding of viral RNA replication and virus - host interactions. Future studies will further exploit DI-RNAs to dissect the mechanism of virus replication, recombination and the role of the host as well as compare similarities between DI-RNAs and helper viruses.

## 6. Conclusions and Future Prospects

Existence of DI-RNAs associated with the infectious parent viruses is one of the perplexing examples of molecular diversity and parasitism. DI-RNAs have a complex relationship with the helper virus and they affect most aspects of virus biology. The relationship between the DI-RNA and the helper virus includes competition for viral- and host factors, induction/suppression of host antiviral responses, and modulation of disease symptoms caused by the helper virus. It will be interesting to explore how DI-RNAs are maintained in the natural viral infections. What advantages do the helper viruses or the host have in co-existing with the DI-RNAs? It is plausible that they might help limit the extent of damage caused by viral infections to their hosts, so that viruses do not kill their hosts as rapidly, thus prolonging the time a host can participate in spreading viruses. As importantly, DI-RNAs are used as surrogate templates in viral replication studies that have contributed to our understanding on the biology of viral infections. DI-RNAs of TBSV have also facilitated the identification of host factors affecting TBSV replication as well as RNA recombination. Many of the *cis*-acting elements for replication were also discovered using DI-RNAs. Several of the above findings with DI-RNAs were subsequently validated with infectious TBSV in the natural plant host. These advances indicate that DI-RNAs are highly evolved molecules that possess authentic *cis*-acting replication elements and utilize helper viral- and host factors for their multiplication. Future experiments will likely dissect the detailed mechanisms of DI-RNA formation and evolution during viral infection. The roles of host and environmental factors will also be studied to throw light on how viruses and sub-viral particles co-evolve in the context of their environment.
